# Editorial: From Apicomplexan Genes to Drug Discovery

**DOI:** 10.3389/fcimb.2021.798754

**Published:** 2021-11-10

**Authors:** Gerhard Wunderlich, Christian Betzel, Carsten Wrenger

**Affiliations:** ^1^ Department of Parasitology, Institute of Biomedical Sciences, University of São Paulo, São Paulo, Brazil; ^2^ Department of Chemistry, Institute of Biochemistry and Molecular Biology, University of Hamburg, Hamburg, Germany

**Keywords:** drug development, machine learning, malaria, mode of action (MOA), *Plasmodium*

Apicomplexan parasites have a significant impact on human and livestock health. The most important apicomplexan parasite is *Plasmodium*, the pathogenic agent of malaria. Malaria is a devastating and quite often deadly parasitic disease that causes substantial public health problems in tropical regions. Due to the high mutational rate of apicomplexan pathogens and their resulting rapid adaptation to environmental changes, or inadequate treatment, drug resistance to the standard and conventional medication is increasing. Therefore, continuous discovery of novel drug targets and the development of new chemotherapeutic agents are essential. In this Research Topic *“From Apicomplexan Genes to Drug Discovery”* we were focussing primary on malaria drug discovery. Several authors have contributed papers to this Research Topic, and an overview of the scientific content is summarized below.

van Heerden et al. demonstrated in their original article machine learning (ML) algorithms to predict the possible mode of action (MoA) of novel compounds. The early identification of the MoA can accelerate hit prioritization, hit-to-lead optimization, and preclinical combination studies in malaria research. Therefore, the authors applied ML on chemically-induced transcriptional profile to identify biochemical pathways on which a compound interferes and thereby signposts a possible interaction partner and the MoA. They assessed different ML approaches and found that only about 50 biomarkers were required to stratify compounds with similar MoA and define chemo-transcriptomic fingerprints for each compound. Appropriate ML tools will facilitate the discovery of new drug targets and will support rapid identification of the MoA and of the novel not yet characterized compounds in the future.

As commented in the review article on antimalarial drug discovery approaches by Rangel and Llinás in this special issue, chemotherapeutics are a major tool to cope with apicomplexan diseases; however, drug resistance against almost all antimalarials have been observed so far, which also includes the artemisinin-based combination therapies. New strategies to discover novel drugs and ideally irresistible drug targets and drug treatments to prevent the rapid formation of resistance in apicomplexan parasites in the near future are urgently needed and are the objective of this Research Topic.

One novel druggable target is proposed by Sumam de Oliveira et al. They highlight targeting post-translational modifications (PTMs) in order to interfere with the control of cellular activities. SUMOylation is one of the respective PTMs which is triggered by the sequential action of three enzymes: E1-activating, E2-conjugating, and E3 ligase. The authors propose *Pf*SUMO as a new potential drug target due to the fact that SUMOylation regulates fundamental biological processes, e.g., gene expression regulation and oxidative stress response in the malaria parasites. Therefore, the development of new chemotherapeutics against this regulation process might interfere with parasites’ proliferation.

The review article by Barra et al. is focussing on another promising drug target, the vitamin B6 biosynthesis pathway in *Plasmodium*. This pathway presents ideal drug target characteristics due to the fact that it is relatively well conserved in bacteria, fungi, and plants but absent in humans, and therefore drug discovery approaches are intended not to harm the human host. In *Plasmodium*, the pathway consists of two enzymes, i.e., Pdx1 (PLP synthase domain) and Pdx2 (glutaminase domain). The authors discuss the unique three-dimensional structures of Pdx1 and Pdx2 and their complexity. For this, they utilized crystallographic data and solution scattering data to exploit the information for the structure-based design and optimization of new substrate analogs that could serve as inhibitors or even act as suicide inhibitors.

In summary, diseases caused by apicomplexan pathogens, such as the malaria parasite, are responsible for several devastating human diseases. Effective vaccines are currently not available, and therefore the treatment of these infectious diseases solely depends on chemotherapeutics. In this context and as outlined before, drug resistance against almost all medications is increasing, and there is concern that this may spread rapidly to other regions. Therefore, novel tools, techniques, and strategies are urgently required to cope with the reducing efficacy of chemotherapeutics against apicomplexan parasites and to identify new drug targets to develop respective new drugs for future applications.

## Author Contributions

CW wrote the manuscript, CW, CB, and GW edited the manuscript. All authors contributed to the article and approved the submitted version.

## Conflict of Interest

The authors declare that the research was conducted in the absence of any commercial or financial relationships that could be construed as a potential conflict of interest.

## Publisher’s Note

All claims expressed in this article are solely those of the authors and do not necessarily represent those of their affiliated organizations, or those of the publisher, the editors and the reviewers. Any product that may be evaluated in this article, or claim that may be made by its manufacturer, is not guaranteed or endorsed by the publisher.

